# Synthesis of 3D Porous Cu Nanostructures on Ag Thin Film Using Dynamic Hydrogen Bubble Template for Electrochemical Conversion of CO_2_ to Ethanol

**DOI:** 10.3390/nano13040778

**Published:** 2023-02-20

**Authors:** Farnood Rahmati, Negar Sabouhanian, Jacek Lipkowski, Aicheng Chen

**Affiliations:** Electrochemical Technology Centre, Department of Chemistry, University of Guelph, 50 Stone Road East, Guelph, ON N1G 2W1, Canada

**Keywords:** electrocatalysis, nanomaterials, dendritic Cu, electrodeposition, CO_2_ reduction, Ag thin film, bimetallic catalysts

## Abstract

Cu-based nanomaterials have been widely considered to be promising electrocatalysts for the direct conversion of CO_2_ to high-value hydrocarbons. However, poor selectivity and slow kinetics have hindered the use of Cu-based catalysts for large-scale industrial applications. In this work, we report on a tunable Cu-based synthesis strategy using a dynamic hydrogen bubble template (DHBT) coupled with a sputtered Ag thin film for the electrochemical reduction of CO_2_ to ethanol. Remarkably, the introduction of Ag into the base of the three-dimensional (3D) Cu nanostructure induced changes in the CO_2_ reduction reaction (CO2RR) pathway, which resulted in the generation of ethanol with high Faradaic Efficiency (FE). This observation was further investigated through Tafel and electrochemical impedance spectroscopic analyses. The rational design of the electrocatalyst was shown to promote the spillover of formed CO intermediates from the Ag sites to the 3D porous Cu nanostructure for further reduction to C_2_ products. Finally, challenges toward the development of multi-metallic electrocatalysts for the direct catalysis of CO_2_ to hydrocarbons were elucidated, and future perspectives were highlighted.

## 1. Introduction

The rapid increase in atmospheric greenhouse gas concentrations has sparked research into the development of highly active and selective electrocatalysts for the electrochemical conversion of CO_2_ [1,2]. The operation of a typical CO_2_ catalyst system involves the electrochemical reduction of dissolved CO_2_ at the cathode, with a balancing oxidation reaction at the anode, which is often the electrolysis of water to O_2_ [3]. In aqueous media, oxygen is the only product generated during the anodic reaction (referred to as OER); however, the cathodic products often undergo multiple intermediate reactions that depend on multiple key factors related to the catalyst properties (e.g., composition, surface morphology, chemical nature) [4,5]. To date, promising catalysts have been shown to generate C_1_ products (e.g., CO, HCOO^-^) with a high level of activity and selectivity, which has further motivated research into the development of catalysts that enable the direct and scalable reduction of CO_2_ to more valuable fuels (C_2_ and C_2+_) [6,7,8]. Within the transition group elements, Cu is the only known element that has the capacity to directly electro-catalyze CO_2_ into multi-carbon products. Consequently, this has attracted considerable interest within the CO_2_ reduction reaction (CO2RR) research field over the last decade [7,9].

The reduction of CO_2_ to C_2_ and C_2+_ hydrocarbons via heterogeneous catalysts is heavily dependent on the interactions between reactants or intermediate products, as well as the active sites of catalysts (i.e., *CO) [1,4,10]. For the initiation of reactions, adequate binding energies must be present to prevent the premature desorption of intermediates, while allowing further reduction toward higher-order hydrocarbons. Rossmeisl et al. described a computational study on the effects of binding energies for adsorbed *H and *CO (Δ*E*_CO*_ and Δ*E*_H*_) on transition group catalysts, and identified Cu as a unique transition group metal with favorability toward *CO and undesirable *H binding energies [11]. Conversely, the weak binding energies of adsorbed *CO that are often found in Au and Ag group metals promote the early desorption of CO2RR intermediates, which generates CO as the main product. Notably, stronger catalytic binding energies that impede the further reduction of CO_2_ to CO translate to active site poisoning through the accumulation of CO, which leads to the domination of competitive HER and poor catalytic performance [12,13]. These previous studies were critical in facilitating a further elucidation of the underlying kinetics that enable Cu to produce C_2_ and C_2+_ hydrocarbons when utilized as a CO2RR catalyst.

While Cu-based catalysts can reduce CO_2_ to value-added products, a major bottleneck toward the practical application of such electrocatalysts is their poor selectivity for higher-order hydrocarbons. Thus, numerous approaches have been investigated to potentially enhance the intrinsic catalytic attributes of Cu to enable their commercialization [14,15,16,17]. Although several Cu-based catalysts have demonstrated promising performance and product selectivity for formic acid or CO, catalyst deactivation and instability due to prolonged activity has slowed the scalability and practicality of Cu-based catalysts for CO2RR [2]. Over the last two decades, advances in high-resolution instrumentation, computational chemistry, and the novel synthesis of nanostructured materials have enabled the development of significantly enhanced catalysts with high surface area-to-volume ratios [18]. As a result, CO2RR research has shifted to the exploration of novel nanostructured electrocatalyst designs to further exploit the improvements enabled through the modification of catalyst surfaces, which often lead to enhanced catalytic performance [19].

Three-dimensional (3D) nanostructures have been the topic of great interest in the recently reported literature on sensors, catalysis, and photovoltaics [20,21,22,23]. These structures are often synthesized by sequential growth using 2D templates, or 3D templates via a single-step growth process [24]. Dynamic Hydrogen Bubble Templating (DHBT) has been employed to generate hydrogen gas in situ as a soft template for the simultaneous reduction of metal ions, which leads to 3D hierarchical porous nanostructures. This process involves three steps, namely nucleation of H_2_ gas at the electrode surface, in situ growth of the bubble, followed by desorption from the surface. As such, the pore morphology obtained from this technique is dependent on the stability of the surface-adsorbed H_2_ bubbles, where the source and concentration of H^+^, substrate material, and the presence of surfactants and additives may dictate the formed morphology. These factors may be adjusted to tune the bubble size before liberation from the substrate. The use of templates with in situ gas evolution for surface nanostructuring allows for the precise control of film thickness, porosity, pore diameter, and dominant facets. Under an applied overpotential, the hydrogen gas generated on the catalyst surface aggregates and briefly adheres to the surface prior to desorption into the gas phase. The simultaneous reduction of metal ions in the precursor solution results in targeted deposition around the short-lived adhered hydrogen bubbles, which leads to the bottom-up growth of a hollow porous framework endowed with a very high surface area [25,26]. The resulting nanostructures exhibit enhanced electrochemical performance due to the greater availability of active sites, higher surface roughness, and abundance of low-coordinated sites (e.g., steps, edges) [23]. As such, various techniques are being investigated to further stabilize and optimize this unique synthesis strategy to be applied as a practical framework for high-performance CO2RR.

Various studies have shown that the derivatives of Cu-based porous structures augmented the catalytic reduction of CO_2_, in contrast to polycrystalline Cu foils [20,21]. Palmore et al. reported on the synthesis of Cu-based nanostructures using dynamic hydrogen bubbles, which had a preference for HCOOH with a Faradaic Efficiency (FE) of 29%, in contrast to <1% observed at a polycrystalline Cu foil [27]. Oxide-derived (OD) Cu catalysts were analyzed by Dutta et al., with an optimal C_2_ efficiency of 55% for the combination of C_2_H_4_ and C_2_H_6_ that was attributed to the improved availability of (100) facet sites, which were proposed as the influencing factor for their selectivity [4]. Further investigations into the tuning of Cu-based porous structures through the use of secondary metals (Au, Ag, Zi, Sn, etc.) were also described by Kottakkat et al. Notably, the simultaneous bimetallic electrodeposition of Ag and Cu ions decreased the overpotential for CO production [28].

For this study, a class of 3D Cu-based porous nanostructures was systematically designed and evaluated for the electrochemical reduction of CO_2_ in a nearly neutral pH electrolyte, with a specific focus on the time and applied current density required for deposition. Further, the optimized deposition parameters in the first phase of this study were used in conjunction with a sputtered Ag thin film to maximize the utility of the 3D porous structure, where the spillover effects of CO intermediates at Ag sites could be harvested to target additional reduction at the pore walls of available Cu sites. While the FE of 33% achieved for HCOOH showed enhanced selectivity on a Cu foam catalyst, the modified catalyst with Ag exhibited remarkable selectivity for the generation of ethanol with an FE of 35% at −1.0 V vs. RHE. The electrochemical kinetics of the Cu/Ag nanostructures was investigated through the impedance (EIS) and Tafel analyses to gain further insights into the enhanced selectivity and activities of the novel catalyst.

## 2. Experiments and Methods

### 2.1. Electrode and Electrolyte Preparation

A pristine Cu sheet was cut into 1 × 1 cm^2^ plates, with each plate spot welded to a pure Cu wire to allow connectivity with electrochemical instruments. Subsequently, the as-prepared substrates were coated with a chemically inert two-part epoxy (3M™ Canada) to insulate all exposed surfaces except for the 1 cm^2^ Cu face. The samples were then thoroughly washed in an acetone bath and sonicated for 20 min, followed by rinsing with isopropyl alcohol and MilliQ water to eliminate any industrial coatings and oil-based agents. Next, the substrates were mechanically polished using a MicroPolish Alumina-impregnated felt pad (0.05 µm, Buehler) to a mirror finish. Finally, the samples were chemically etched in a 2 M nitric acid solution for 180 s, rinsed thoroughly, and dried under a stream of Ar gas prior to use in the synthesis procedure.

To assess electrochemical performance of the catalysts, a solution of 0.1 M potassium bicarbonate (KHCO_3_) was freshly prepared before each experiment. The electrolyte solutions were initially purged with Ar gas and then purged continuously with the reactant gas (CO_2_) until a pH of 6.8 was obtained, indicative of CO_2_-saturated 0.1M KHCO_3_ electrolyte. To assess the activities of the catalyst in the HER regime, 0.1 M of potassium sulfate (K_2_SO_4_) solution was employed as the electrolyte to assess the hydrogen evolution (HER) activity, with a balanced pH of 6.8 using sulfuric acid (H_2_SO_4_).

### 2.2. Electrochemical Measurements

Voltammetry and impedance spectroscopy experiments were conducted using a potentiostat (SI 1287A, Solartron) equipped with an impedance analyzer system (Model 1260A, Solartron), which were configured in parallel. CorrWare and ZPlot software packages were utilized for data collection and processing. For all experiments, the positioning of counter and reference electrodes remained constant to ensure minimal deviations. EIS analyses were performed in the frequency range between 1.0 MHz and 1.0 mHz, with an amplitude of 10 mV. To fit the Nyquist plots, The ZView software package was employed to fit the Nyquist plots. For all measurements, an Ag/AgCl (3.0 M KCl) (CHI 111, CH Instruments) was utilized as the reference electrode (RE), while a custom-made Ti/Ta_2_O_5_-IrO_2_ with a surface area of 3 × 1 cm^2^ was used as the counter electrode (CE) for boosted stability. All recorded potentials in this study were converted to the Reversible Hydrogen Electrode (RHE) reference scale according to the following equation:

(1) E (vs. RHE)=0.197 V+0.059 V×pH+E (vs. Ag/AgCl)

### 2.3. Product Characterization

To characterize the generated gaseous products, the cathode chamber of the H-cell was continuously purged in-line to a sensor-equipped (TCD and FID) gas chromatography (GC) system (Multiple-gas-#5, SRI Instruments), which allowed for the detection of carbonaceous products, as well as hydrogen in the fluent stream. A customized gas-tight Teflon H-cell was employed for the product analysis. At the conclusion of the experiments, the electrolyte in the cathodic chamber was immediately collected and sealed for further characterization and analysis using a ^1^H NMR spectrometer (600 MHz Bruker Avance III NMR, Bruker). A 350 µL volume of the catholyte was added to a 350 µL internal reference of 0.05 wt.% 3-(trimethylsilyl)propionic-2,2,3,3-d_4_ acid sodium salt in D_2_O.

To measure the total amount of the liquid-phase products generated from CO2RR, a chemical oxygen demand (COD) analysis was also conducted using 174-334 accu-TEST standard range (5–150 mg L^−1^) twist fcap vials, where the same catholyte sample batch for NMR was introduced to a vial that contained a chromic acid solution [29,30]. After the reaction, an assessment was performed using a portable spectrophotometer (HACH-DR 2800) operated at a 420 nm wavelength. For COD analysis, the formed aqueous hydrocarbon compounds undergo oxidation according to the following:

(2) $$C_{a}H_{b}O_{c}+\left(a+\frac{b}{4}-\frac{c}{2}\right)O_{2}\to a\;CO_{2}+\frac{b}{2}H_{2}O$$

where *a*, *b*, and *c* refer to the stoichiometric ratio of carbon, hydrogen, and oxygen, respectively, in the collected products. For each oxygen molecule, four electrons were transferred following the equation below:

(3) $$O_{2}+4H^{+}+4e^{-}\to 2H_{2}O$$

As a result, the total consumed charge (*Q_COD_*) for the present carbonaceous products could then be calculated using the following equation:

(4) $$Q_{COD}=COD\left[\frac{mg}{L}\;O_{2}\right]\times \left(\frac{4FV}{32000}\right)$$

where *F* and *V* represent the Faraday constant and the volume of the solution used, respectively.

## 3. Results and Discussion

### 3.1. Fabrication of Cu Foam under Different Deposition Times

In a time-controlled deposition study, Cu foams were created using a solution containing Cu^2+^ ions under an applied constant current with varying deposition times in a two-electrode electrochemical system. A solution containing H_2_SO_4_ (200 mL, 2 M) and CuSO_4_∙5H_2_O (200 mL, 0.1 M) was prepared at a 1:1 volumetric ratio to give final concentrations of 50 mM CuSO_4_ and 1.0 M H_2_SO_4_. A customized H-cell with two chambers separated by a glass frit was filled with 25 mL of the solution, followed by 25 min of continuous purging with Ar gas. This ensured that *HER* and Cu reduction were the primary reactions in this system under an applied potential.

A DC power supply (E3634A, Keysight Technologies) was calibrated with a constant current (CC) program for different deposition times. Following preparation, the Cu samples were positioned in the cathodic chamber, and the electrochemical deposition proceeded under a constant current for 10, 30, 50, 80, and 100 s (Table S1). Under an applied voltage, hydrogen gas was vigorously generated and desorbed from the surface. The formation of observable bubbles close to the cathode surface provided a soft 3D template for the deposition of Cu.

### 3.2. Fabrication of Cu Foam under Different Deposition Currents

To systematically identify the optimal deposition current for the highest CO_2_ reduction performance, the total charge transfer for the highest-performing time-controlled sample was retained to assess the effects of the applied current on the electrochemical performance. The highest-performing sample was synthesized based on 80 C of charge transfer (Table S2). As such, 80 C was used to calculate the deposition time at 200, 400, 600, 800, and 1000 mA to retain the total charge transfer under different currents. The measured voltages to apply the target currents were 4.3 V, 6.7 V, 9.6 V, 10.6 V, and 12.1 V, respectively. The as-prepared Cu foam samples were gently soaked in a MilliQ water bath, followed by drying in a stream of Ar gas at ambient room temperature.

### 3.3. Synthesis of Ag-Modified Cu Foam

To introduce a secondary metal at the lowest level of the 3D porous Cu structure, a 200 nm Ag thin film was deposited onto the pretreated Cu substrate using a magnetron. The coated 1 cm^2^ sample was then insulated following the same procedure described above. Next, the Cu nanostructures were grown onto the Ag-modified substrate using the optimized deposition parameters derived from the results obtained thus far. The sputtered layer was quantified at a thickness of ~200 nm atop the initial Cu substrate, which ensured adequate structural integrity to prevent delamination during the catalytic reactions.

### 3.4. Surface Characterization

In a typical bottom-up electrochemical deposition nucleation and growth system, the thickness of the grown film is closely correlated with the duration of the applied potential, a trend that is expected to be observed under different deposition lengths in the synthesis of Cu nanostructures. This relationship deviates from linearity as a function of the deposition time, where the in operando increase in surface area dynamically reduces the applied current density. As a result, the reconstruction of surficial nanostructures can easily occur, which affects the availability of active sites. Chorkendorff et al. reported on an electropolished porous Cu-based structure that had an FE of 14% for C_2_H_4_ and 5% for CH_4_ [31]. Similarly, Palmore et al. described the use of a soft-template porous Cu, which yielded formate as its main product with an FE of 29% [27]. As such, a comprehensive investigation of the influences of deposition parameters on the catalytic activities of this catalyst subgroup might reveal the range of different products reported in the literature.

For our first analysis, nanostructured Cu samples were grown to examine the direct impacts of the electrodeposition time and current on the CO_2_ reduction performance. The SEM images of mirror-finished Cu substrate were provided for comparison (Figure S1). The as-prepared samples were also imaged using an FE-SEM to characterize the surface nanostructures and investigate the morphological variations due to changes in the studied parameters (i.e., deposition time and current). Considering the time-dependent samples, low magnification imaging revealed that the surface level structures were not uniform across the entire sample until 50 s of deposition (Figure S2). Additionally, an increase in the deposition time to 80 s demonstrated the presence of a uniform surface structure across the entire substrate. Notably, it was observed that further increases in the deposition time yielded larger pore diameters, as well as pore wall thicknesses. High magnification SEM images revealed dendritic growth, predominantly for the sub 50 s deposition times. Longer deposition times exhibited a shift toward the anisotropic densification of the initial dendritic branches, which increased the pore wall thickness (Figure S3).

The catalysts underwent vertical scanning interferometry (VSI) to obtain quantitative measurements of the porous structures by profiling their surface structures, which enabled the precise 2D elucidation of their physical properties. In addition, the optical roughness factor was calculated using the root mean squared (RMS) average between the height deviations and the mean surface area using the following equation:

(5) $$R_{q}=\sqrt{\frac{1}{MN}\overset{N}{\underset{j=1}{\sum }}\overset{M}{\underset{i=1}{\sum }}Z^{2}\left(x_{i},y_{j}\right)}$$

where *M* and *N* are the dimensions of the scanning area in the *X* and *Y* pixel positions, for which *Z* height values are optically measured.

According to Figure S3, the measured film thickness and, alternatively, pore depth, demonstrated a linear relationship with deposition time (up to 80 s). Additional deposition time (to 100 s) yielded a negligible increase in pore depth, which suggested a diminishing rate of Cu ion deposition. This was due either to insufficient molarity, dominating the HER as the result of a much larger available surface area, or the shift toward the densification of dendritic branches rather than layered vertical growth [27]. Additionally, an analysis of the roughness factor obtained through VSI depicted an increasing trend in parallel with the deposition times. This suggested the formation of new layers atop initial structures, which hinted at the densification and prolonged growth of the dendritic nanostructures that comprised the pore walls. Topological maps of the time-controlled samples revealed that the pore radii steadily increased with longer deposition times of up to 80 s; however, further deposition had the effect of reducing them.

The SEM images of the samples deposited for different durations (Figure S4) suggested that the microstructured morphologies were dependent on the applied current. Initial observations revealed that the porous structures were highly ordered and repeatable across the surface of the sample. High magnification SEM showed that lower currents produced nanostructures with rough and stepped edges, while increased currents resulted in round and softer corners/edges within the pore walls.

**Figure 1:**
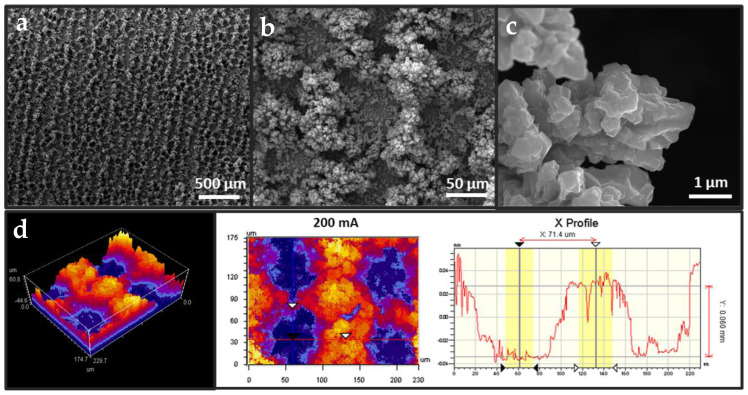
(**a**–**c**) SEM imaging of current-controlled *f*-Cu/Cu sample deposited at 200 mA, at magnifications of 100×, 1000×, and 20,000×, respectively. (**d**) 3D optical model and topological map of the as-deposited *f*-Cu/Cu sample via VSI.

Considering the deposition mechanism of a bottom-up growth approach, the applied current dictated both the dynamic rate of template formation and metal ion deposition alongside mass-transport limitations. While the deposition rate of metal ions initially reached diffusion limitations, in operando increases in the surface area and the reduction in localized current densities stabilized hydrogen bubbles to form larger aggregates, leading to larger pores over prolonged deposition times or under higher currents. VSI analysis of the samples revealed a backward trend in surface roughness and pore depth (Figure S5). As seen in Figure 1, the synthesis of samples under a 200 mA deposition current showed the largest R_q_ and a pore depth of 60 µm, both of which were reduced by increasing the deposition current. Further, it was proposed that higher deposition currents contributed significantly to the HER, thereby potentially sterically impeding the availability of Cu^2+^ ions at the double layer. A summary of the VSI analysis of the pore profiles can be found in Table S3. As revealed through roughness and pore depth analysis, the rapid generation and aggregation of hydrogen at the cathode surface influenced the creation of the smoother edges and steps observed under larger applied currents. Among all the samples deposited under different currents, the radii at the bottoms of the pores remained at ~40 µm.

**Figure 2:**
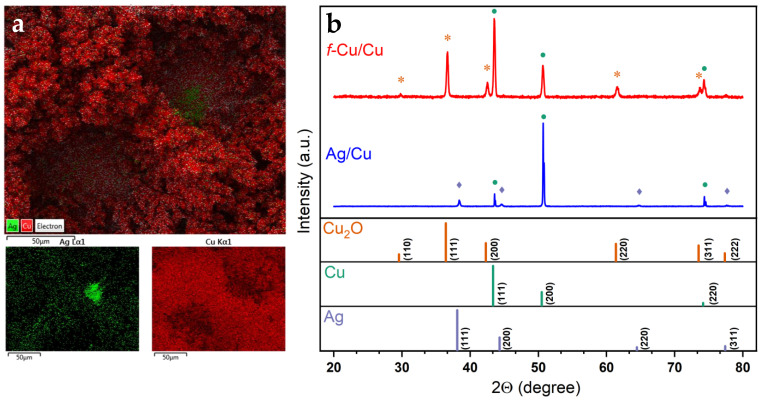
(**a**) EDX analysis of *f*-Cu/Ag/Cu depicting Ag sites present at the bottom of the 3D *f*-Cu porous nanostructure. (**b**) Ex situ x-ray diffractograms of as-deposited *f*-Cu_200mA_/Cu and Ag/Cu, with standards obtained through JCPDS database for Cu_2_O (05-0667), Cu (04-0836), and Ag (04-0783). Peaks are labeled with symbols corresponding to the standard JCDPS reference for (*) Cu2O, (●) Cu, and (◊) Ag.

To validate the presence and stability of the Ag thin film throughout the deposition process, energy dispersive spectroscopy (EDS) was employed to obtain the elemental map of the pores. Figure 2a displays the EDS mapping of the *f*-Cu/Ag/Cu sample, showing the elemental composition of two candidate pores, confirming the Ag sites present at the bottom of the 3D *f*-Cu porous nanostructure. XRD was utilized to characterize the crystallinity of the as-prepared *f*-Cu nanostructures. As shown in Figure 2b, major reflections were detected at angles of 29.7°, 36.7°, 42.6°, 61.5°, and 73.7°, which were indexed to the copper (I) oxide planes of (110), (111), (200), (220), and (311), along with reflections at 43.5°, 50.7°, and 74.3°, which were indexed to the metallic Cu planes of (111), (200), and (220), respectively. Data for the Cu_2_O (*05-0667*), Cu (*04-0836*), and Ag (*04-0783*) were obtained from the JCPDS database and simulated for comparison. The XRD fingerprints of all investigated samples were identical for the most part, and exhibited outstanding facets between the various parameters tested. Figure 2 displays the crystallographic spectra of the *f*-Cu sample, showing numerous surface oxide (111) and (200) facets. As these data were acquired ex situ, oxide formation during transport was unavoidable; thus, the detection of copper oxides was largely attributed to this factor. Furthermore, the XRD pattern of the Ag-modified Cu substrate exhibited peaks for both Cu^0^ and Ag. Similarly, metallic Cu^0^ was also found on the as-deposited *f*-Cu sample extracted from the polycrystalline Cu substrate as a powder. Due to the high overpotential deposition, less stable facets converged toward more stable facets through reconstruction. Overall, a range of crystalline facets was detected on the as-deposited Cu nanostructures, with a larger number of (111) and (200) that corresponded to other dominant Cu facets.

### 3.5. ECSA and DLC

**Figure 3:**
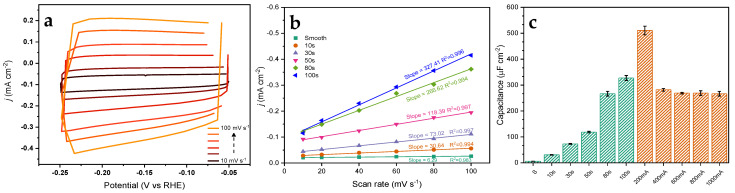
(**a**) DLC cyclic voltammetry for an 80 s time-controlled sample, (**b**) plot of current density versus scan rate for DLC derivation for time-controlled samples. (**c**) Comparison of DLC of all test samples plotted against normalized smooth polished Cu foil substrates (time and current controlled samples were shown as green and red colors, respectively).

The electrochemically active surface area (EASA) may be obtained by measuring the double-layer capacitance (DLC) when performing cyclic voltammetry in a purely capacitive (non-faradaic) voltage region. The impacts of electrodeposition parameters on the EASA were explored through scan rate-dependent cyclic voltammetry in a non-faradaic potential window (Figure 3a). The capacitance of the double layer was then approximated by linear fitting of the current density against the scan rate. Subsequently, the capacitance values were normalized against a known smooth finished surface to correlate the capacitance to quantitative surface roughness values (Figure 3b), in contrast to the geometric analyses performed by SEM and VSI. A summary of all DLC measurements is found in Table S4.

The EASA analysis of time-dependent *f*-Cu electrodes revealed an increase in the electrochemically active surface area with longer deposition times, which agreed with the geometric trend measured via SEM (Figure S2). A mirror-finished Cu plate was utilized as the reference roughness with a double-layer capacitance of 6.29 µF cm^−2^. Compared to the as-deposited sample prepared under a 30 s deposition time, a 10× increase in the EASA was measured through this method, followed by a 50x increase when 100 s had elapsed. These measurements aligned with the optical measurements and calculations obtained by VSI. The roughness observed through VSI analysis was also confirmed by these measurements (Figure S6). A 200 mA deposition current demonstrated the highest capacitance at 510.6 µF cm^−2^ and a surface roughness (RMS R_q_) of 30.0 µm cm^−2^, which yielded an 80x increment in the availability of active sites compared with pristine Cu. A further increment in the deposition current lowered the capacitance, which was also confirmed through optical surface measurements using VSI. Topological and EASA studies suggested that the deposition time was the core parameter to influence the availability of surface sites between the fabricated samples. While maintaining the total charge transfer at 80 coulombs, a higher deposition current was correlated with shorter deposition times, and demonstrated depreciating EASA. As a visual reference, VSI models of time-controlled samples were compiled to generate a brief video that illustrated bottom-up growth with various deposition time windows (Figure S7).

### 3.6. Electrochemical Reduction Performance Analysis

#### 3.6.1. Oxide Passivation Layer

The electrodeposition of Cu ions generates a highly reactive Cu surface where spontaneous oxidation can occur immediately upon exposure to air or disconnection from a bias source. As the reduction of the surface oxide layer during CO2RR can skew efficacy measurements, the catalysts were subject to a pretreatment protocol to normalize the passivation layer prior to CO_2_ reduction tests. In the recent literature, the intentional oxidation of Cu catalysts (often referred to as ‘oxide-derived’) has demonstrated potential improvements in selectivity to make C_2_H_4_ and C_2_H_6_. However, some studies have reached conflicting conclusions as to the influence and stability of oxide species at high overpotentials, where it is thought that most surficial oxide-derived species undergo in situ reduction prior to the catalysis of reactants. Huang et al. reported on a study where an initial oxide mass contribution of 10% from Cu_2_O was reduced to 5% following prolonged CO_2_ electrolysis [32]. Further work by Dau et al. demonstrated that surface oxide species were highly unstable and mostly reduced to Cu^0^ prior to the onset potential for CO_2_ reduction (~0.7 V vs. RHE) [28].

While the presence of surface oxides is unavoidable in reactive samples, the ambiguity of surface oxides between samples must be considered. As such, the prepared samples underwent a second-stage pretreatment cycle to ensure that the surface oxides between samples were normalized. Cyclic voltammetry (20 cycles) in the potential range below the reduction of CO_2_ at a scan rate of 100 mV s^−1^ was performed prior to the CO_2_ reduction experiments. Pretreatment cycling was conducted under the same conditions as CO2RR to enable CO_2_ reduction experiments without the need to exchange electrochemical vessels.

#### 3.6.2. CO2RR Performance

To benchmark the electrocatalytic performance of the synthesized catalysts, linear sweep voltammograms (LSVs) were performed in a solution containing a CO_2_-purged 0.1 M KHCO_3_ electrolyte with a final electrolyte pH of 6.8. LSVs were also performed in an Ar-purged 0.1 M K_2_SO_4_ to evaluate the catalytic performance under favorable HER conditions. The current profile obtained from the two systems was then used to roughly approximate the expected faradaic efficiency (Figures S8–S10).

The LSV experiments showed an increase in catalytic activities with longer deposition times of up to 80 s. In contrast, the 100 s deposition time sample (*f*-Cu_100s_) performed at a lower current density than those with shorter deposition times (Figure 3). While mass transport limitations may limit high surface area catalysts, in this case, higher currents were achieved with the 80 s deposition sample, which confirmed higher mass transport headroom in this system. However, these limitations were further amplified in 3D matrices where the availability of protons could be prevented due to the undesirable diffusion pathway between the double layer and active internal pore sites.

Further, comparisons of the onset potentials for CO_2_ reduction between the deposition time-controlled samples revealed that they decreased as a function of the deposition time (Figure S8). While pristine Cu foil showed a CO_2_ reduction onset of −0.8 V vs. RHE, the 80 s deposition time had an onset potential of −0.2 V, which differed significantly from the pristine sample. Notably, however, the *f*-Cu_100s_ sample deviated from this observed trend by showing a more negative CO_2_ reduction onset potential than the other samples.

An analysis of all time-controlled samples revealed a strong preference for the 80 s deposition time, as this sample exhibited the highest currents and lowest CO_2_ reduction onset potentials. Therefore, 80 s was determined as the first optimized input for our systematic approach to assess additional parameters. As such, 80 coulombs was used as the optimized total charge transfer for the fabrication of current-controlled *f*-Cu electrodes. Through the analysis of the fabricated samples thus far, an optimal charge transfer of 80 coulombs was demonstrated to show the highest catalytic performance. As such, this value was used to resolve a range of deposition currents to investigate the influence of current density on the reduction performance of the catalyst.

**Figure 4:**
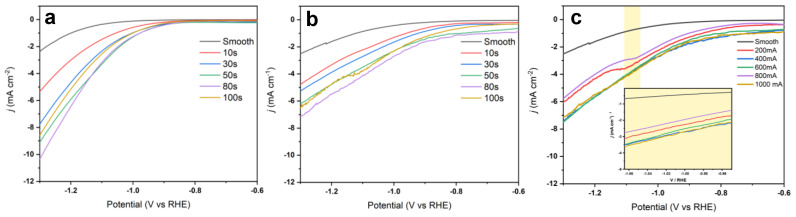
LSV plots of time and current-controlled *f*-Cu/Cu samples measured in (**a**) Ar-saturated and (**b**,**c**) CO_2_-saturated 0.1 M KHCO_3_ electrolytes.

Under the constant applied current studied in the second stage, the maximum charge transfer was locked to the same coulombs for 80 s deposition (80 coulombs), which resulted in a sample with the following parameters: 200 mA for 400 s, 400 mA for 200 s, 600 mA for 134 s, and 800 mA for 100 s. Figure S9 displays the electrochemical performance of the current-controlled samples in the CO_2_ reduction setup. Initial observations showed slight differences between the samples when compared to the time-controlled samples. While the measured HER remained constant across the tested samples, the CO_2_ reduction performance was shown to have a deviation of ~700 µA when tested under −1.0 V vs. RHE. The current-dependent samples exhibited relatively equivalent catalytic performance (Figure 4). The obtained LSVs under CO_2_-purged conditions exhibit a noisier current profile at higher applied overpotentials. This behavior might be caused by the generation and liberation of gas products. It is worth noting that poor diffusion of generated products away from the active sites present within the 3D porous structure might further impede the stable electrochemical process. Additionally, a shoulder profile is observed near −1.09 V vs. RHE in current controlled samples, which was attributed to in situ blockage of surface sites due to generated gaseous products remaining on the catalyst surface.

The 200 mA deposition current sample demonstrated the highest electrochemically active surface area and surface roughness (RMS R_q_) among all the tested samples. Therefore, this sample was employed to further assess the performance stability and further optimization. It is well understood that the nanostructuring of surfaces dramatically increases the EASA, which results in increased current within the same geometric area. In 3D samples such as the electrodeposited *f*-Cu, the matrix of pores developed through the HER provided a unique architecture for the engineering of bimetallic structures. Specifically, it was concluded that the available area at the bottom of this 3D hierarchical structure could be further enhanced to systematically target early desorbed intermediates such as CO to synergistically re-catalyze at Cu sites along the cylindrical structure. While the synergistic spillover effect is not yet fully understood, one-pot tandem catalysis systems can be designed to benefit from the selectivity of various transition metals to overcome monometallic catalysis bottlenecks, specifically premature desorption of CO_2_ reduction intermediates. With this perspective, the unique incorporation of a secondary metal (Ag) to promote the desorption, re-adsorption, and further reduction of intermediates by the primary metal (Cu) was further investigated.

Throughout the analyses and imaging of the as-deposited samples, it was observed that the bottom of the porous structure retained the initial substrate material quite well (Figure 2a). This revelation inspired us to utilize the substrate region of the 3D nanostructure to incorporate a secondary metal, where potential spillover effects could be enhanced due to the 3D structure of the pores. Using magnetron sputtering, a thin film of Ag (~200 nm) was deposited onto the pretreated Cu substrates where the underutilized surfaces available at the bottom of each pore could be potentially endowed to serve as CO generation sites. For this arrangement, the formed CO_2_ reduction intermediates at Ag sites (*CO) were in proximity to active Cu sites, which enabled the further reduction of CO to higher-order hydrocarbons. To the best of our knowledge, this configuration of Ag within 3D porous Cu nanostructures is the first studied case in the literature.

**Figure 5:**
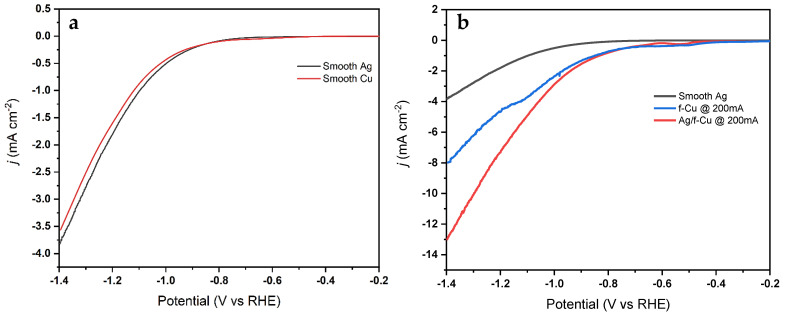
LSV polarization curves of (**a**) smooth Cu and Ag sputtered Cu in Ar purged environment, and (**b**) *f*-Cu_200mA_/Cu and Ag modified *f*-Cu_200mA_/Ag/Cu in CO_2_ purged environment.

As seen in Figure 5, the Ag-modified porous *f*-Cu electrode demonstrated higher catalytic performance when compared to the non-modified Cu samples. Further, the electrochemical activity of the Cu substrate sputtered with Ag was compared against the *f*-Cu_200mA_ and *f*-Cu_200mA_/Ag/Cu. This suggested a synergistic effect in the Ag-modified *f*-Cu_200mA_ sample due to the specific interactions between the sputtered Ag and fabricated porous f-Cu.

Chronoamperometric (CA) experiments were performed under moderate overpotentials prior to the onset of HER in a CO_2_-purged 0.1 M KHCO_3_ electrolyte. The anodic and cathode chambers of the H-cell were separated using a cation exchange membrane (CEM) to enable the analysis of liquid products. Simultaneously, the cathodic chamber was connected to the GC input line, which allowed the gas-phase products to be characterized in operando.

**Figure 6:**
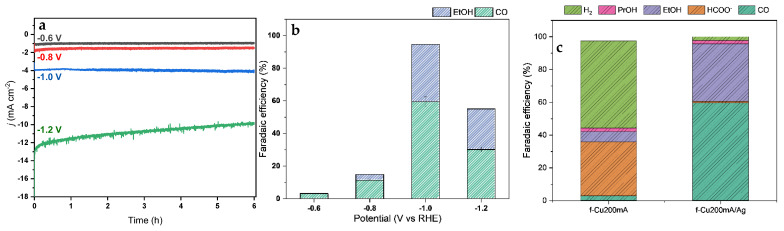
(**a**) Chronoamperometry plot of *f*-Cu_200mA_/Ag/Cu under a CO_2_-saturated 0.1M KHCO_3_ electrolyte. (**b**) Total product analysis of *f*-Cu_200mA_/Ag/Cu. (**c**) Total detected liquid and gas-phase products and corresponding efficiencies of *f*-Cu_200mA_/Ag and *f*-Cu_200mA_.

Chronoamperometry experiments proceeded for six hours to assess the stability of the catalyst under sustained activity (Figure 6). Steady-state currents sustained throughout the runs were achieved within 30 s of the experiment. Notably, a gradual decrease in current density was observed at −1.2 V vs. RHE. SEM images of the working sample prior to and following the chronoamperometry experiments were obtained to probe the stability of the surface nanostructures. The nanometric surface structures were shown to be identical and well intact post-operation (Figure S11).

For all chronoamperometry experiments under different overpotentials, the cathodic chamber was continuously purged with CO_2_, which allowed for the online measurement of gas-phase products via the connected GC system. Subsequently, following the completion of the experiment, the catholyte was immediately stored and sealed for NMR and COD analysis to ensure that no volatile components (i.e., ethanol, acetone) were lost through the sample transfer process (Figure 6b,c). The *f*-Cu_200mA_ electrode primarily generated HCOOH at 33.1% FE, with small amounts of propanol and ethanol at 2.3% and 6.1% FEs, respectively. Hydrogen was the main gaseous product, with minor amounts of CO measured at 2.9% FE. The non-modified porous *f*-Cu_200mA_ was active for single carbon products, which were typically categorized to be generated from a different reaction pathway than that of CO production. Tang et al. recounted an in situ Raman spectroscopy study of HCOOH and CO generation at Cu surfaces, and concluded that the suppression of one pathway resulted in an increase in catalytic activities of the other pathway [31].

In an analysis of the gas-phase products of the Ag-modified *f*-Cu electrode, the sample demonstrated a higher selectivity toward CO versus the non-modified Cu samples, with an FE of 59.5%, with a suppressed H_2_ evolution down to an FE of 2.3% (Figure 6b). Furthermore, an analysis of the catholyte through NMR and COD detected ethanol with a measured FE of 35.2% and formate with an FE of 1%. Additionally, minor concentrations of propanol were measured across the applied potentials, which suggested an independent propanol production pathway through the addition of Ag.

For the Ag-modified Cu nanostructures, it was observed that the generated products differed from the range of Cu samples under study. At −0.6 V vs. RHE, only small concentrations of CO were quantified via in-line GC. An additional overpotential (−0.8 V vs. RHE) yielded the first measured quantity of ethanol. The highest faradaic efficiency for the Ag-modified sample was observed at −1.0 V vs. RHE for CO and ethanol, while an additional increase in overpotential diminished the products and generated significant quantities of hydrogen. This shift to the HER at higher overpotentials was also reported by Ager et al., where the studied oxide-derived Cu sample demonstrated its highest performance at −1.0 V vs. RHE, and imposing an added 100 mV of overpotential reduced the efficiency from 54.5% to 35% [33]. As a result, the study asserted that the severe depletion of accessible CO_2_ at the active sites led to the domination of the HER at higher overpotentials.

COD was used on the same catholyte sample utilized for ^1^H NMR analysis to validate and quantify the generated liquid products that might be difficult to deconvolute from NMR analysis (Figure S12). Both COD and NMR analyses of the catholyte sample yielded the same total FE in generated liquid-phase products. An increment of 4% in FE of total liquid products was obtained at −1.2 V vs. RHE via COD. Furthermore, NMR analysis indicated that small concentrations of propanol (PrOH) were resolved through NMR analysis, but were below the quantification limit. Consequently, the 4% deviance observed in the COD FE was attributed to the presence of propanol, which has been reported for Cu-based CO_2_ catalysis in the literature [5].

At the time of this study, there were limited reports of Cu-based catalysts that directly produced ethanol in near-neutral electrolytes. Our results herein suggest that this unique product pathway was enabled due to the incorporation of Ag within a 3D porous Cu foam to amplify the spillover effect of the important CO intermediate. Extremely large EASA leading to the rapid consumption of H^+^ cations suggested a rapid localized increase in pH, where pH-dependent reaction pathways were uniquely enabled. A recent study by Goddard et al. concluded that CO_2_ reduction at pH 1 involved kinetically suppressed multi-carbon production pathways, where in contrast, a typical intermediate was identified at neutral pH that shared both single and multi-carbon pathways with identical favorability [34]. Their work also concluded that enhanced selectivity for multi-carbon products was observed at pH 12 due to kinetically blocked C_1_ pathways. In our case, the rapid consumption of H^+^ ions may have led to the same effect, where C_2+_ pathways were favored due to an increase in the local pH at the cathode surface. While the effect of the local pH on product selectivity is known to be critical, the addition of Ag within the pores would not impose a pH change locally and in bulk. As a consequence, a more plausible hypothesis for the change in product selectivity toward higher carbon C_2_ products via the incorporation of Ag within *f*-Cu might be attributed to the stabilization of CO intermediates at the Ag-Cu sites. Recent computational DFT studies have demonstrated the C_1_ and C_2_ product pathways were enabled via the stabilization of CO intermediates on an Au surface [35]. Since Au and Ag share a similar CO2RR pathway, it is reasonable to assume that the incorporation of Ag at *f*-Cu 3D porous wells resulted in recapturing or shuffling of Ag-liberated *CO intermediates, thus facilitating further reduction to form EtOH and PrOH.

**Figure 7:**
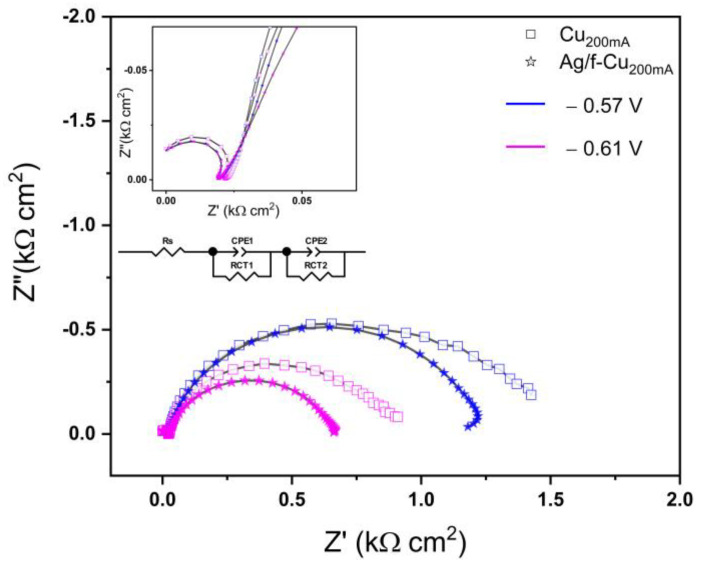
Nyquist plots of *f*-Cu_200mA_/Cu and *f*-Cu_200mA_/Ag/Cu with the displayed equivalent electrical circuit.

To gain additional insights into various reduction pathways observed in this system, electrochemical impedance spectroscopy (EIS) and Tafel plot analysis were performed to assess the adsorption and rate-limiting mechanisms of the catalytic process. The EIS experiments were carried out at the potentials of −0.57 V and −0.61 V vs. RHE. For comparison, the Nyquist plots of the *f*-Cu_200mA_ electrodes with or without the Ag modification are displayed in Figure 7. For clarification, the high-frequency portion of the EIS spectra is enlarged and also presented as an inset in Figure 7. The equivalent circuit used for fitting the EIS data is also included as an inset in Figure 7, where R_s_ refers to the solution resistance. R_CT1_ and R_CT2_ denote the charge transfer resistances associated with the two semicircles of the Nyquist plot, corresponding to the pre-adsorption of the CO_2_ species and the following electrochemical reduction. The fitting results are listed in Table 1, showing that both R_CT1_ and R_CT2_ were decreased with the increase in the cathodic potential from −0.57 to −0.61 V and that R_CT1_ was significantly decreased with the Ag modification. It could be deduced that the incorporation of Ag enhanced the pre-adsorption kinetics of CO_2_, thus providing additional carbon-substrate-linked species where the carbon–carbon (C-C) linking would be promoted via reactant or intermediate shuffling or spillover. Specifically, it can be hypothesized that the formation and availability of additional electroactive species locally at Cu-Ag could be the key factor in promoting C-C linking, thereby favoring the further reduction of CO_2_ (and its intermediates) toward C_1_ and C_2+_ products.

**Figure 8:**
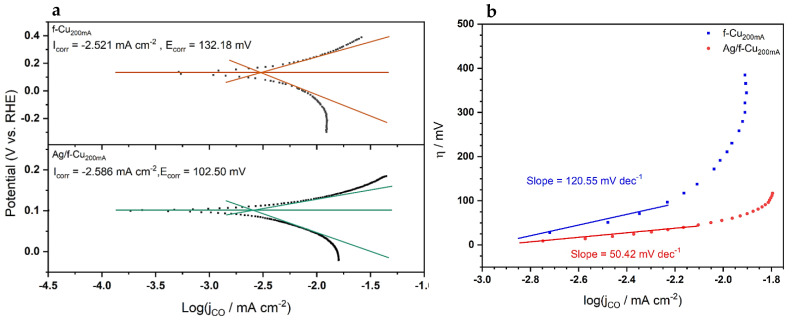
(**a**)Tafel plots of *f*-Cu_200mA_/Cu foam and *f*-Cu_200mA_/Ag/Cu foam, including the linearized cathodic and anodic branches. (**b**) Tafel slopes of *f*-Cu_200mA_/Cu and *f*-Cu_200mA_/Ag/Cu.

Tafel plots of the samples were generated to compare Tafel slopes between the two samples (Figure 8). Tafel analysis demonstrated that the Ag-modified Cu nanostructures exhibited a Tafel slope equal to 50.42 mV dec^−1^, which was appreciably less than the unmodified Cu nanostructures with a Tafel slope equal to 120.55 mV dec^−1^. Generally, smaller Tafel slopes exhibited faster reaction kinetics, which were often correlated with higher catalytic activities. In the recent literature, Tafel slopes were commonly compared against rate expressions to deduce the reaction pathways. Xu et al. [36] recently suggested rate expressions and their related Tafel slopes for an initial activation stage for *CO_2_* reduction reactions at a Tafel slope of 1188 mV dec^−1^:

(6) $$CO_{2}+e^{-}+\;*\;↔{\left(CO_{2}^{-}\;\right)}_{ad}$$

followed by the subsequent reaction of *CO_2_* to *COOH* at 59 mV dec^−1^:

(7) $${\left(CO_{2}^{-}\;\right)}_{ad}+H_{2}O↔COOH_{ad}+OH^{-}$$

and the final reaction step to *CO* at 39 mV dec^−1^:

(8) $$COOH_{ad}+H_{2}O+e^{-}↔CO_{ad}+OH^{-}$$

Analyzing the measured Tafel slopes from our study, the data suggested that the initial activation step was the rate-determining step that bottlenecked the reaction process. A calculated Tafel slope of 120.55 mV dec^−1^ was within the range of error for the literature value of 118 mV dec^−1^, which suggested that the rate-limiting step was the initial activation process of *CO*_2_. In the case of the Ag-modified *f*-Cu, the measured Tafel slope of 50.42 mV dec^−1^ resided between the suggested second and third steps from the literature. While it is challenging to pinpoint the rate-limiting step, the lower Tafel slope, due to the incorporation of Ag, strongly hinted at synergistic interactions between Ag at the bottoms of the pores and the Cu pore walls.

## 4. Conclusions

Nanostructured 3D porous Cu foams were systematically synthesized and analyzed to determine the optimal deposition parameters for a porous Cu catalyst. Further tuning through the novel introduction of Ag within the Cu foam pores was shown to enhance the selectivity and activities of the composite catalyst toward the generation of higher-order C_2+_ products. Electrochemical and characterization analyses via XRD, SEM, and VSI unveiled a 3D porous framework with an extensive electrochemically active surface area. This work reports on a series of Cu-based nanostructured foams that exhibited enhanced catalytic activities, product selectivities, and onset reduction potentials. Analyses of gas and liquid phase products yielded from chronoamperometry experiments detected C_1_ products at an applied potential of −0.8 V vs. RHE, where HCOO^-^ was the main product at 33% FE. The enhanced selectivity and onset potential were studied via EIS and Tafel analyses. While it was reported that C_2_ and C_2+_ reaction pathways were kinetically favorable at pH > 7, the suppression of CH_4_ formation and preference toward HCOO measured through product analysis proposed an inadequate CO intermediate for the formation of C–C coupling. As such, the targeted inclusion of Ag within the cylindrical framework of the Cu foam was proposed to enhance the catalytic performance of the catalyst via the enhanced spillover of CO at Ag active sites. The product selectivity of the Cu foam modified with Ag exhibited a unique preference toward EtOH with the highest achieved faradaic efficiency of 35% at −1.0V vs. RHE. To further understand the increase in selectivity and performance, the EIS and Tafel experiments were performed, which proposed a shift in reaction pathways, as well as a lower charge transfer resistance when Ag was incorporated. The results of this study have demonstrated strong potential for the utilization of porous Cu nanostructures deposited on Ag thin films as a highly selective and tunable electrocatalyst for scalable applications in the electrochemical reduction of CO_2_.

## Figures and Tables

**Table 1:** EIS-fitted parameters for the Cu_200mA_/Cu and Cu_200mA_/Ag/Cu electrodes.

Applied Bias (V)	R_S_ (Ω/cm^2^)		R_CT1_ (Ω/cm^2^)		CPE-T1 (mF cm^−2^)		R_CT2_ (Ω/cm^2^)		CPE-T2 (mF cm^−2^)	
	Cu_200mA_/Ag/Cu	Cu_200mA_/Cu	Cu_200mA_/Ag/Cu	Cu_200mA_/Cu	Cu_200mA_/Ag/Cu	Cu_200mA_/Cu	Cu_200mA_/Ag/Cu	Cu_200mA_/Cu	Cu_200mA_/Ag/Cu	Cu_200mA_/Cu
−0.57	18.75 ± 0.21	22.63 ± 0.32	210.5 ± 21.1	506.2 ± 7.9	25.81 ± 1.03	9.559 ± 0.013	1098.3 ± 6.4	1031.67 ± 51	2.54 ± 0.18	9.94 ± 0.49
−0.61	18.75 ± 0.21	22.36 ± 0.29	108.8 ± 6.5	387.5 ± 3.1	19.24 ± 0.76	6.007 ± 0.012	557.3 ± 4.1	579.8 ± 28	2.38 ± 0.15	6.01 ± 0.24

## Data Availability

The data presented in this study are available on request from the corresponding author.

## Supplementary Materials

The following supporting information can be downloaded at https://www.mdpi.com/article/10.3390/nano13040778/s1. Figure S1: Scanning electron microscope (SEM) images of mirror-finished Cu samples; Figure S2: SEM images of as-deposited *f*-Cu samples under time-controlled deposition for (row A) 30, (row B) 50, (row C) 80, and (row D) 100 s; Figure S3: VSI topological analysis of *f*-Cu fabricated at different times at (A) 30, (B) 50, (C) 80, and (D) 100 s with cross-sectional depth analysis; Figure S4: Scanning electron microscope (SEM) images of as-deposited *f*-Cu samples under current-controlled deposition at (row A) 200 mA, (row B) 400 mA, (row C) 600 mA, and (row D) 800 mA; Figure S5: VSI topological analysis of current-controlled f-Cu fabricated at (A) 30, (B) 50, (C) 80, and (D) 100 s with cross-sectional depth analysis; Figure S6: Plot of current density versus scan rate for DLC derivation of current-controlled samples; Figure S7: Stitched 3D models of as-deposited time-controlled samples to visualize impacts of time on growth structures; Figure S8: (left) LSV plots of f-Cu samples synthesized under time-controlled deposition. (right) Approximated Fes from differences in Ar (red) and CO_2_ (grey)-saturated electrolytes; Figure S9: (left) LSV plots of f-Cu samples synthesized under current-controlled deposition. (right) approximated Fes from differences in Ar (red) and CO_2_ (grey)-saturated electrolytes; Figure S10 LSV plots of f-Cu200mA/Ag sample tested under CO_2_ (red) and Ar (grey) conditions; Figure S11: SEM images f-Cu200mA/Cu sample (a,b) before and (c,d) after chronoamperometry stress test; Figure S12: Comparison of detected liquid product analysis of f-Cu200/Ag sample measured through NMR and COD techniques; Table S1: Parameters for synthesis of the tested time-controlled *f*-Cu samples, with measured current density and corresponding efficiencies; Table S2: Parameters for synthesis of the tested current-controlled f-Cu samples, with measured current density and corresponding efficiencies; Table S3: Summary of all double layer capacitance values measured through steady-state cyclic voltammograms. Roughness factors are normalized against mirror-finished smooth Cu plate of 1 × 1 cm^2^ geometric surface area; Table S4: Summary of all double layer capacitance values measured through steady-state cyclic voltammograms. Roughness factors are normalized against mirror-finished smooth Cu plate of 1 × 1 cm^2^ geometric surface area.
