# Supplementary material for: Synthesis of 3D Porous Cu Nanostructures on Ag Thin Film Using Dynamic Hydrogen Bubble Template for Electrochemical Conversion of CO2 to Ethanol
Source: Nanomaterials (Basel). 2023 Feb 20;13(4):778. doi: 10.3390/nano13040778 (PMC9959227; doi:10.3390/nano13040778)
Supplement: Supplementary file 1 [file nanomaterials-13-00778-s001.zip › nanomaterials-2102777-supplementary.pdf]

Supplementary Materials

Synthesis of 3D Porous Cu Nanostructures on Ag Thin Film using Dynamic Hydrogen Bubble Template for Electrochemical Conversion of CO_2 to Ethanol

Farnood Rahmati, Negar Sabouhanian, Jacek Lipkowski* and Aicheng Chen*

Electrochemical Technology Centre, Department of Chemistry, University of Guelph
50 Stone Road East, Guelph, Ontario N1G 2W1, Canada

***Correspondences: jlipkows@uoguelph.ca (J.L.); aicheng@uoguelph.ca (A.C.)

Chemicals and Materials

All chemicals used for preparing the precursor and electrolyte solution were purchased and used without further purification. Copper (II) sulfate pentahydrate (99.999%, $CuSO_4 \cdot 5H_2O$), sulfuric acid (99.999%, H_2SO_4), perchloric acid (70%, $HClO_4$), potassium bicarbonate (99.7%, $KHCO_3$), and potassium sulfate (99.99%, K_2SO_4) were purchased from Sigma Aldrich. Carbon dioxide (99.999%, CO_2) and Argon (99.995%, Ar) gas cylinders were purchased from Praxair. All water used for preparation and dilution was purified through water purification system (Direct 8, Milli-Q®). Strips of high-purity 110-grade Copper (99.99%) were purchased from McMaster-Carr.

Surface Characterization

The morphologies of the electrochemically synthesized catalysts were characterized by field emission scanning electron microscopy (FE-SEM, FEI Quanta FEG 250 SEM) equipped with a Schottky source at 20 keV bias, and images were acquired at a working distance (WD) of 10.0 mm. The compositional analysis of the samples was measured through another SEM (FEI Inspect S50) equipped with an energy-dispersive X-ray spectrometer (EDX) attachment, using AZtech Spectrum software. Crystallography data was collected using an X-ray diffractometer (XRD, PANalytical Empyrean) in a reflection geometry with Ni-filtered CuK_α radiation source and PIXcel1D linear detector in the 20-80º 2θ, collected and processed using X'pert Data Collector and HighScore Plus software, respectively. Topographical analysis of the catalysts was done using vertical scanning interferometry (VSI, Wyko NT3300 Optical Profiler) through 0.5x FOV and 50x Objective lens, for a total 26.9x magnification.

Gas Product Analysis

To ensure accurate measurement of the gas products, if the steady state current density was below 5 mA cm^{-2}, the gas products were analyzed offline through port syringe injection, while higher current densities were analyzed online and offline. For our configuration, the TCD was used to measure the H_2 gas, while the FID was used to quantify all carbonaceous gases in the product stream. Furthermore, both detectors were calibrated using a standard mixture of gases in Ar to derive an eight-point calibration curve. The quantified gases were used to calculate the respective Faradaic efficiencies using the following equation:

$$\boldsymbol{FE_g = \frac{2n_g \cdot F}{Q_{total}} = \frac{2PVx_gF/RT}{\int_0^t Idt}}$$

where n_g is the amount of gas generated during the electrolysis time t (unit: mol); F is the Faraday constant (96485 C·mol^{-1}); Q is the integrated charge (unit: C); V is the volume of the headspace (20.0 mL); x_g is the faction of gas g detected by GC; P and T are ambient pressure (101 kPa) and temperature (298 K), respectively; R is the gas constant (8.314 J·mol^{-1}·K^{-1}); I is the current of the chronoamperometry curve.

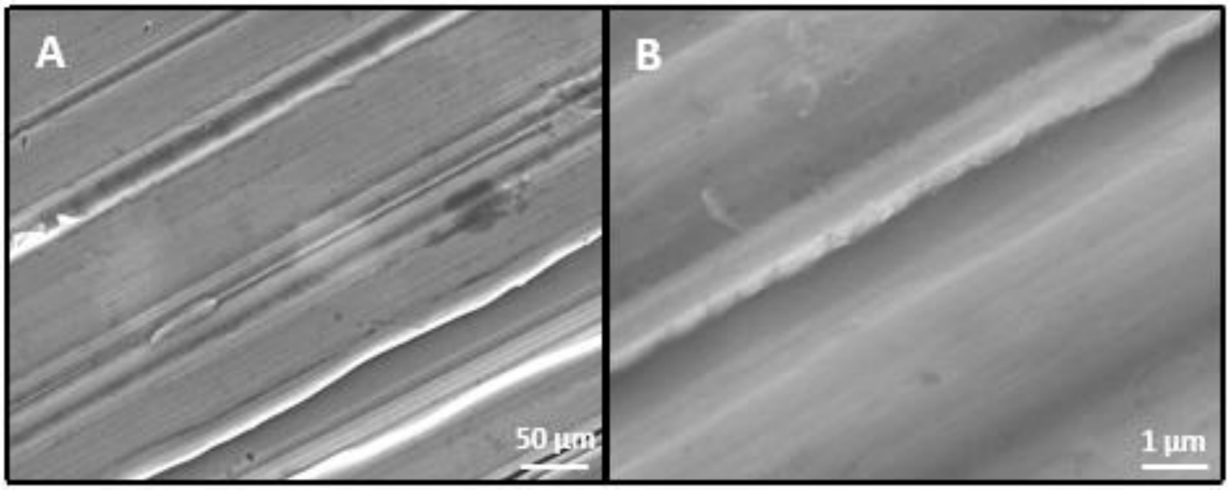

Figure S1. Scanning electron microscopic (SEM) images of the polished Cu substrate.

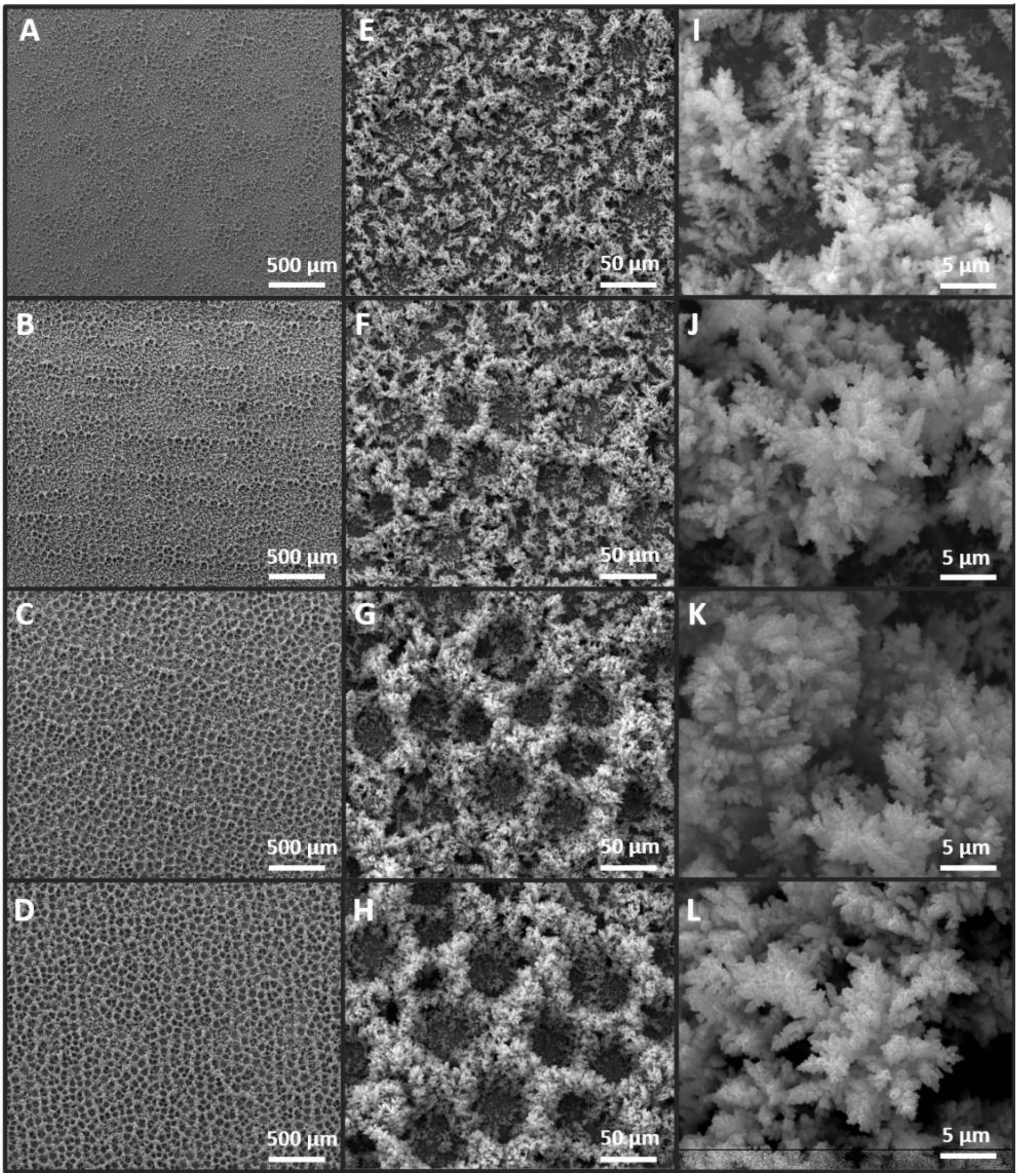

Figure S2 SEM images of as-deposited *f*-Cu samples under time-controlled deposition for (row A) 30, (row B) 50, (row C) 80, and (row D) 100 seconds.

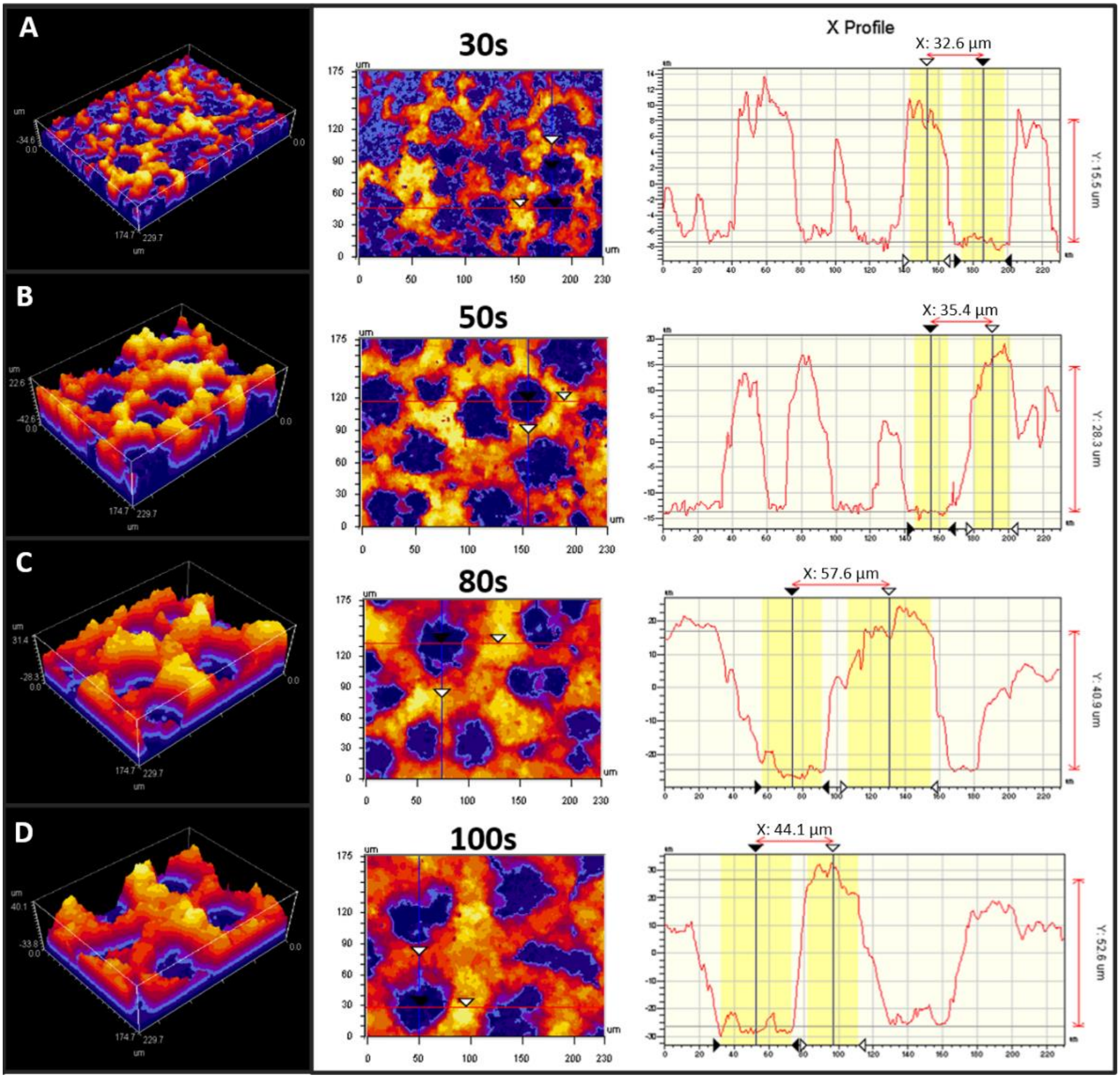

Figure S3 VSI topological analysis of *f*-Cu fabricated at different times at (A) 30, (B) 50, (C) 80, and (D) 100 seconds with cross-sectional depth analysis.

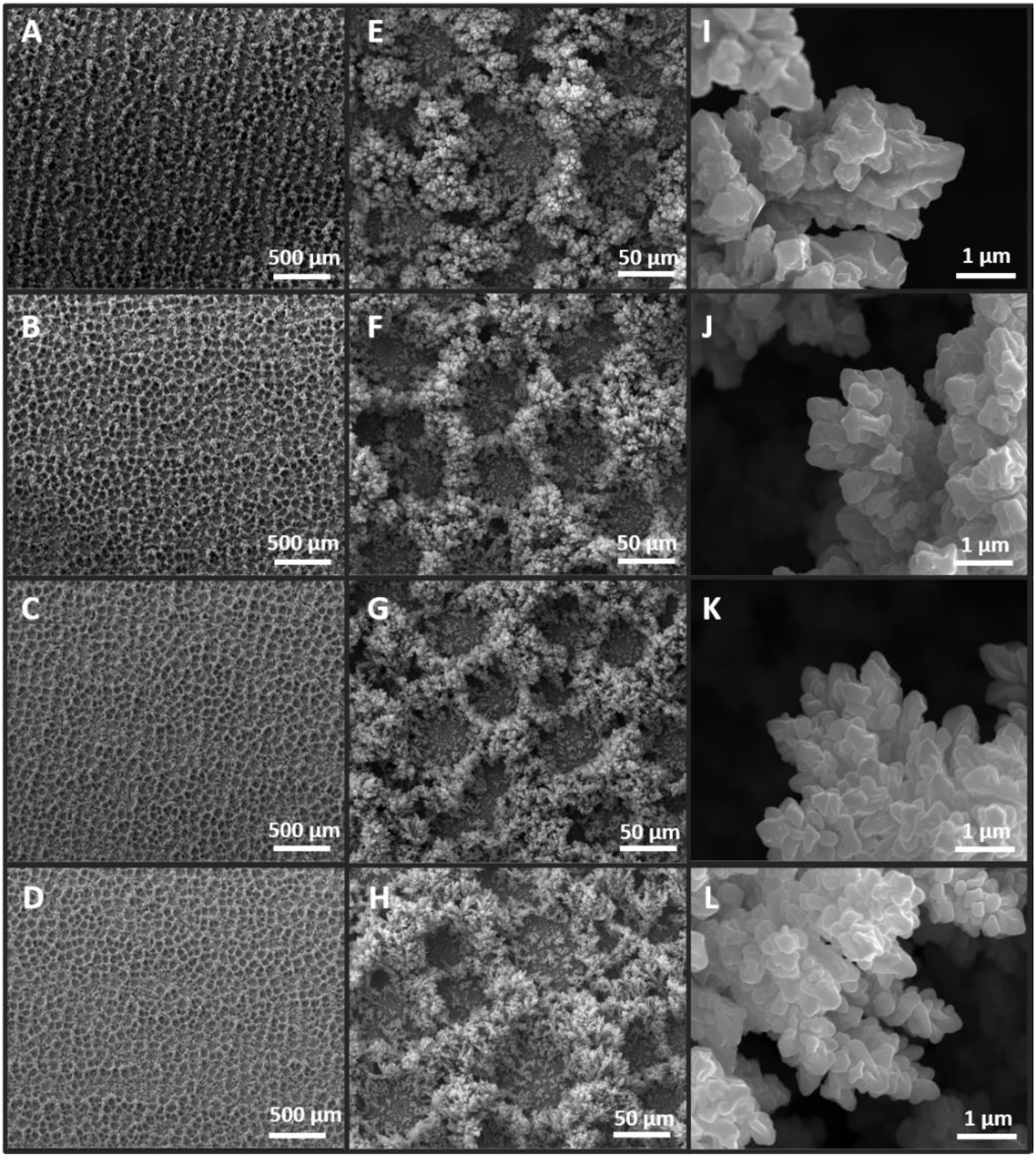

Figure S4 SEM images of as-deposited *f*-Cu samples under current-controlled deposition at (row A) 200 mA, (row B) 400 mA, (row C) 600 mA, and (row D) 800 mA.

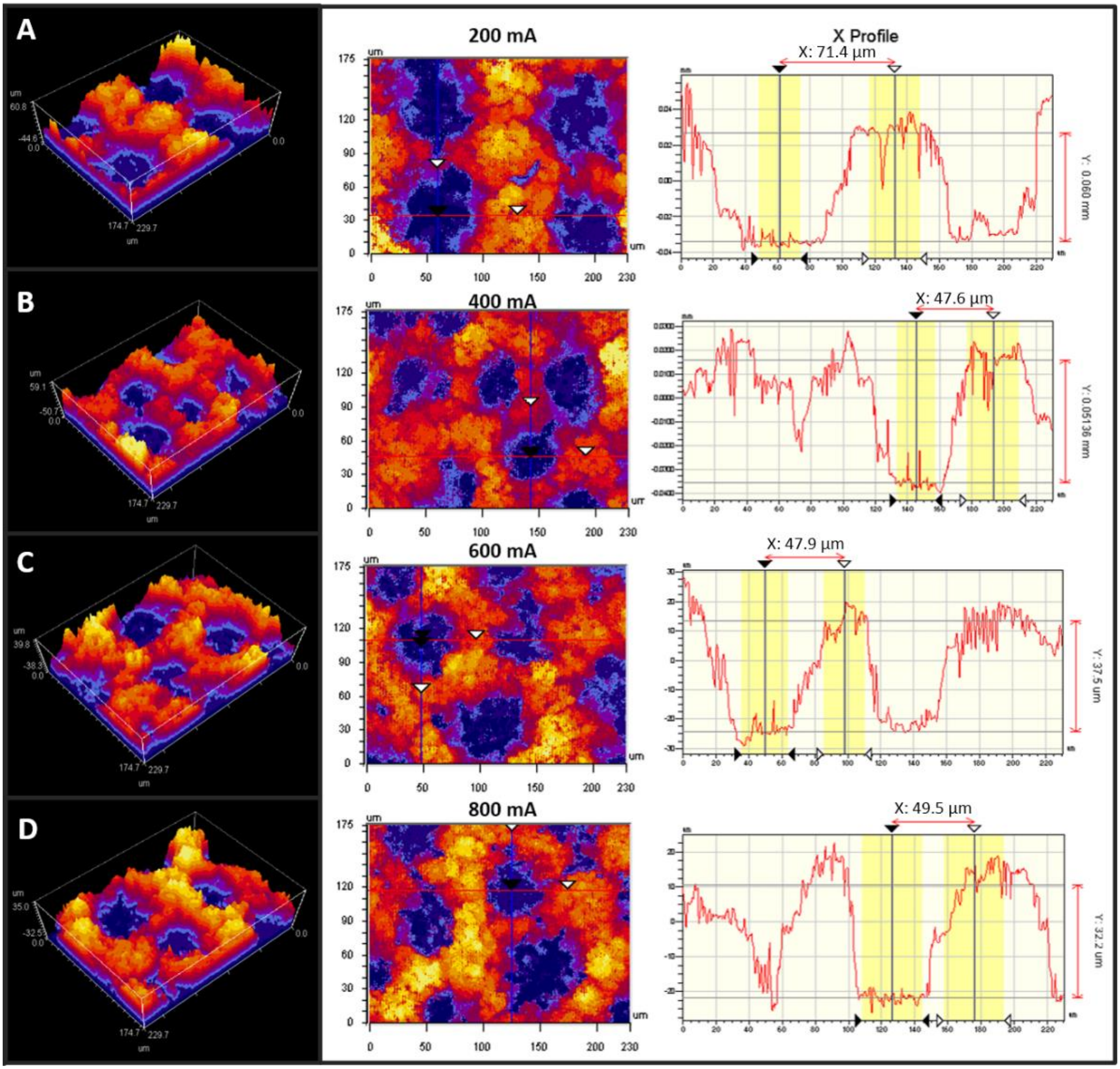

Figure S5 VSI topological analysis of current-controlled *f*-Cu fabricated at (A) 30, (B) 50, (C) 80, and (D) 100 seconds with cross-sectional depth analysis.

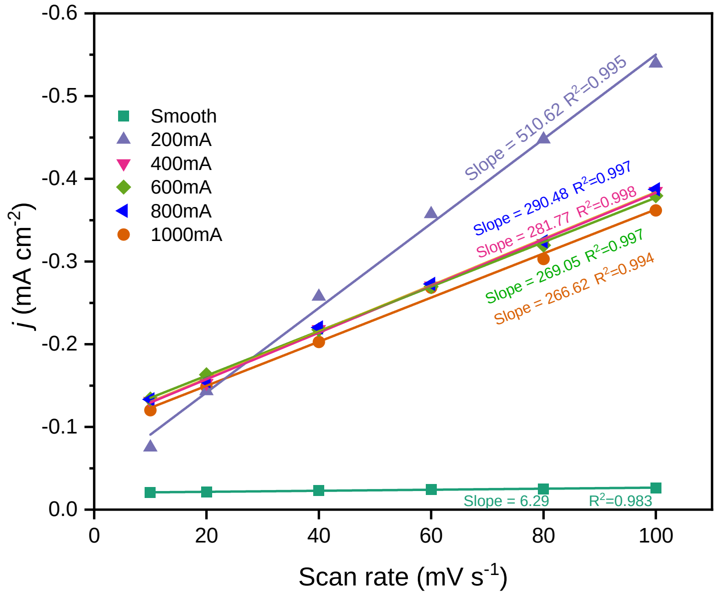

Figure S6 Plot of current density versus scan rate for DLC derivation of current-controlled samples

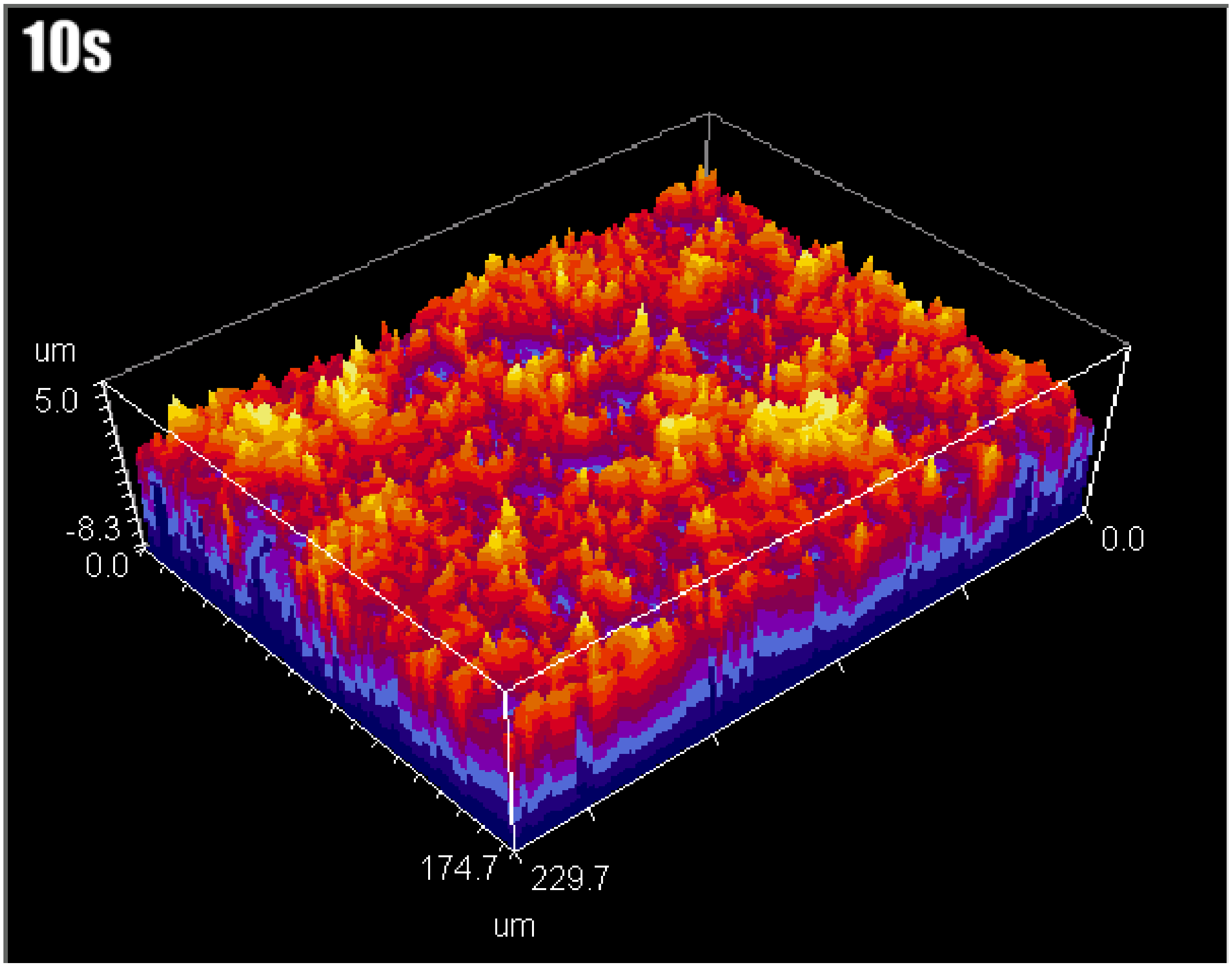

Figure S7 Stitched 3D models of as-deposited time-controlled samples to visualize impact of time on growth structure.

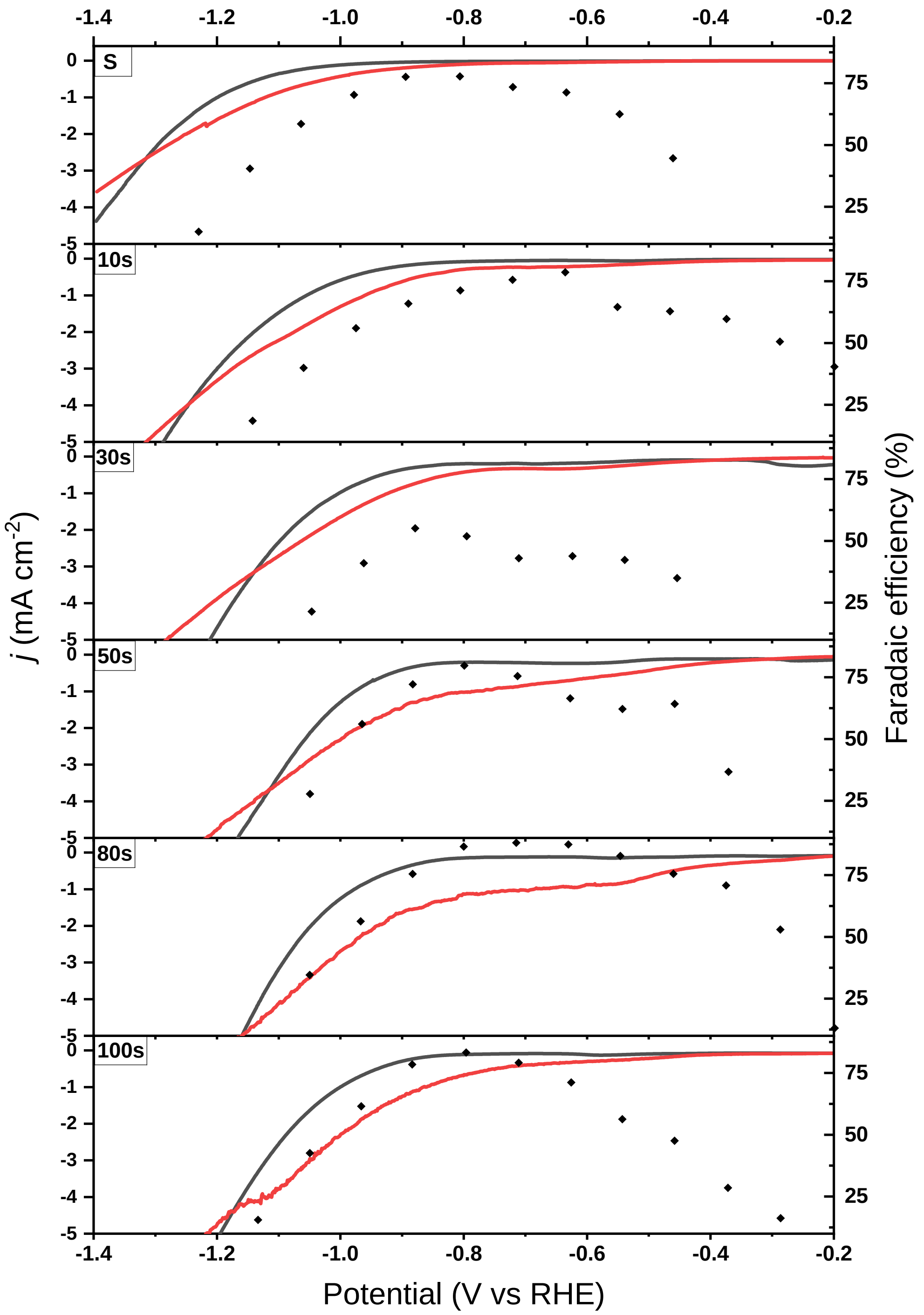

Figure S8 LSV plots of *f*-Cu samples synthesized under time-controlled deposition recorded in the Ar (grey) and CO_2 (red) saturated electrolytes. The dots represent the calculated current efficiency at different potentials.

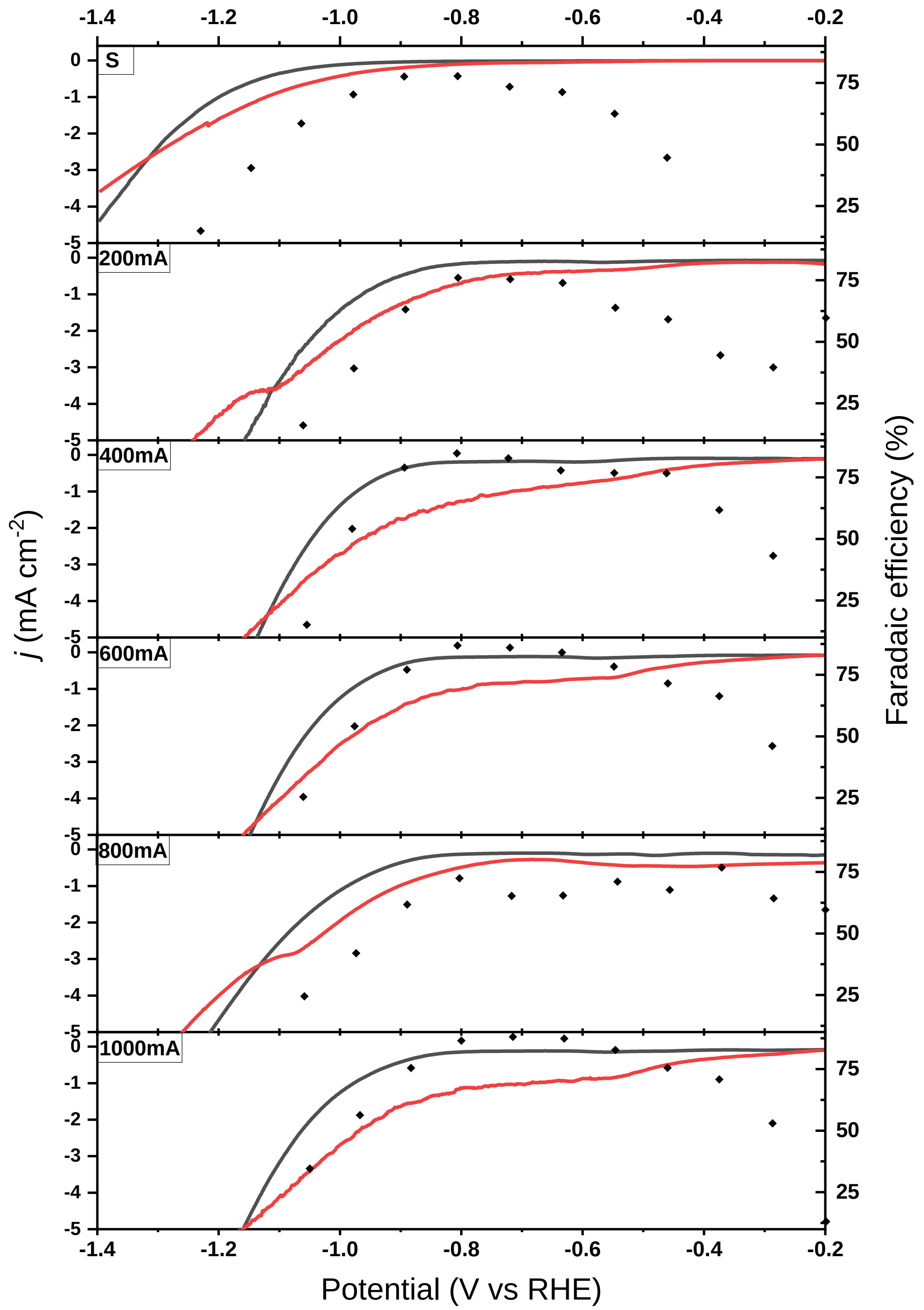

Figure S9 LSV plots of *f*-Cu samples synthesized under current-controlled deposition recorded in Ar (grey) and CO_2 (red) saturated electrolytes. The dots represent the calculated current efficiency at different potentials.

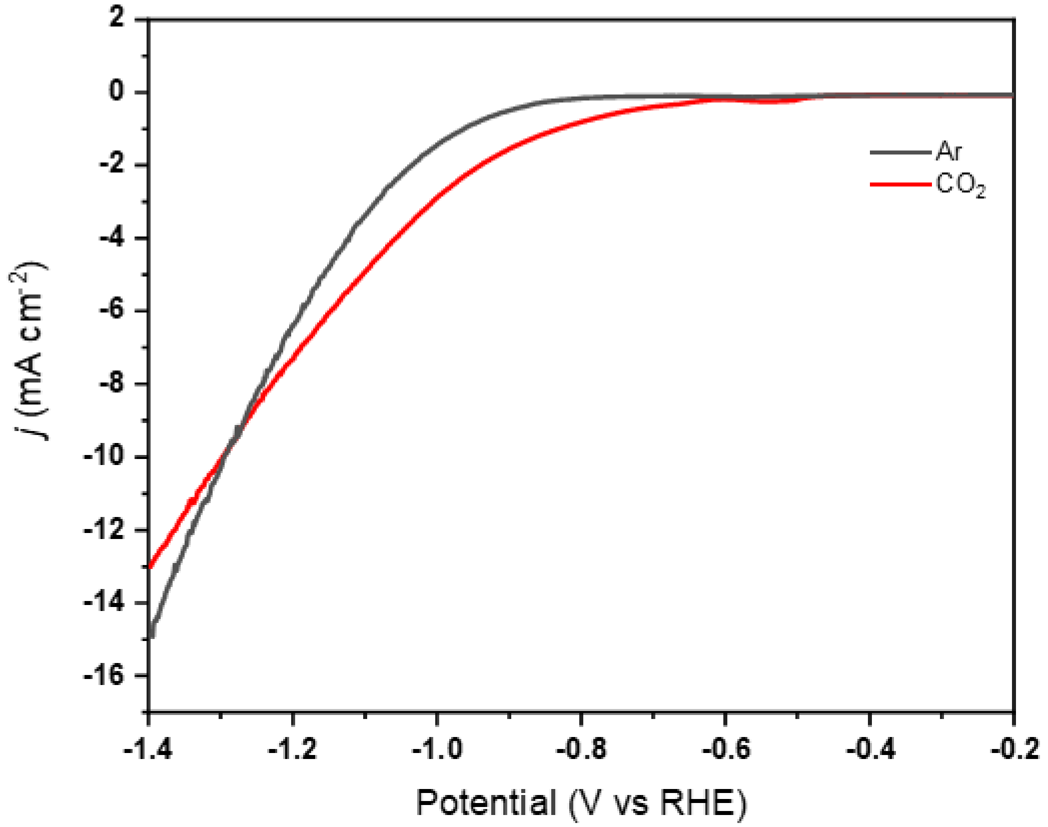

Figure S10 LSV plots of *f*-Cu_{200mA}/Ag sample recorded in CO_2 (red) and Ar (grey) saturated 0.1 M $KHCO_3$ electrolyte.

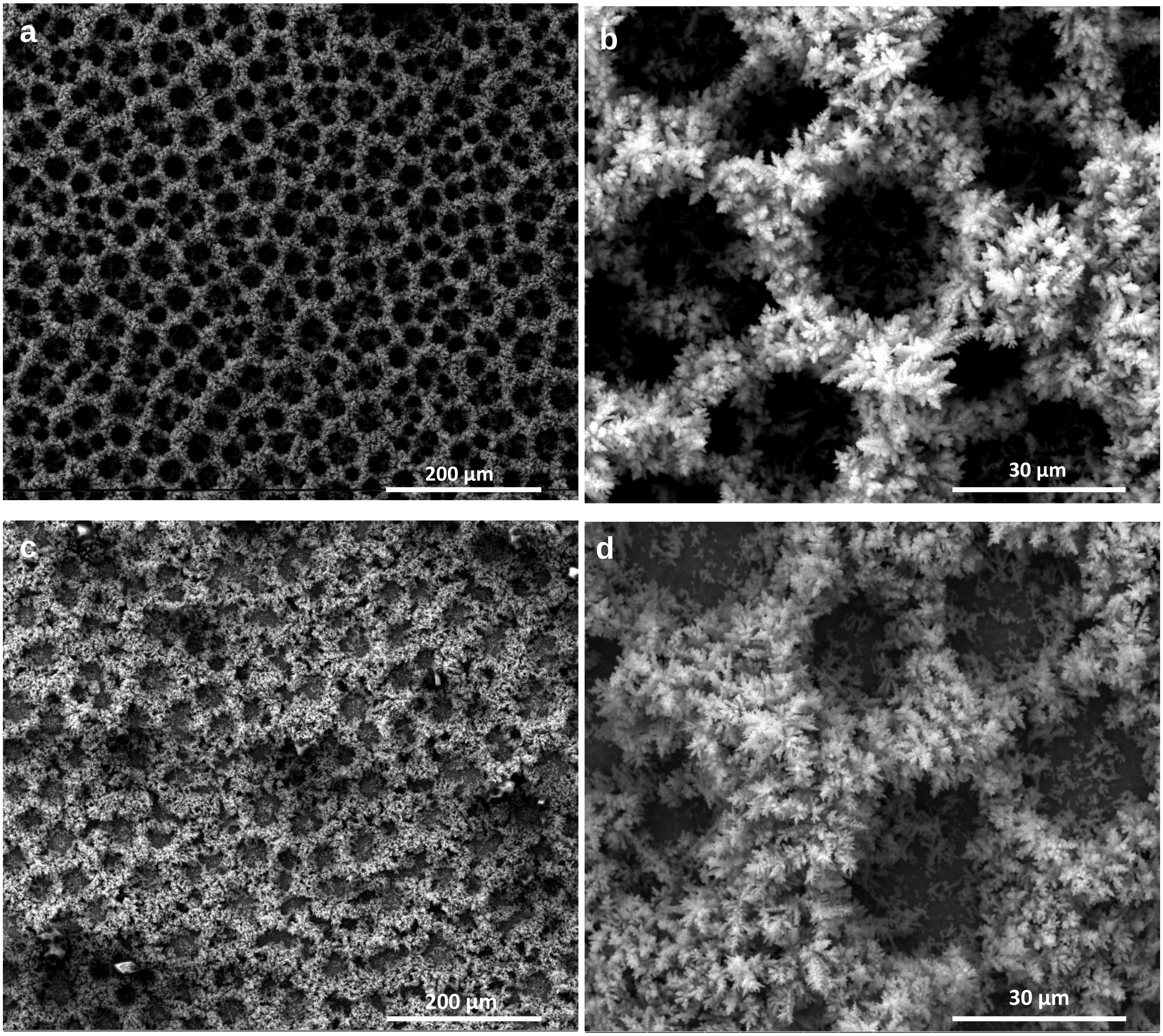

Figure S11 SEM images *f*-Cu_{200mA}/Cu sample (a,b) before and (c,d) after the chronoamperometric test.

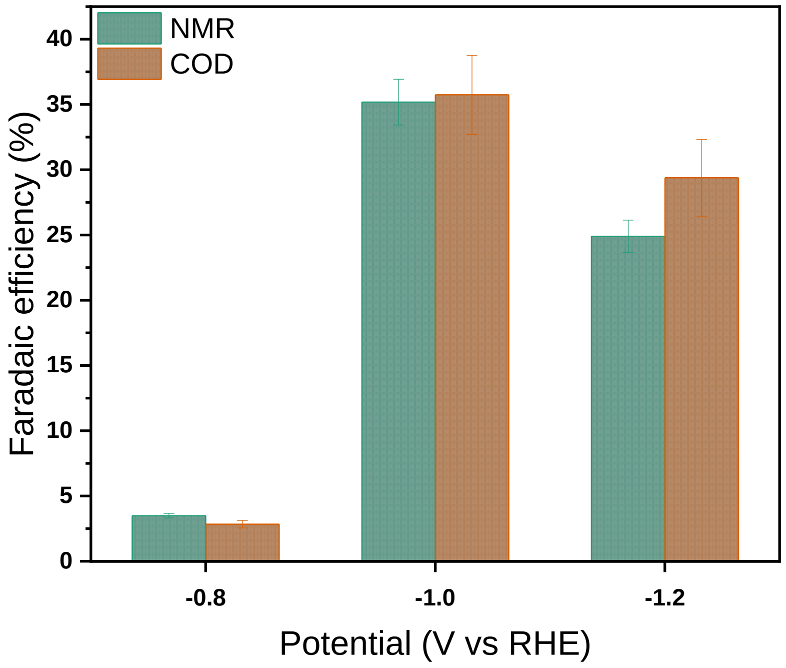

Figure S12 Comparison of the liquid products of the f-Cu_{200}/Ag sample measured through NMR and COD techniques.

Table S1 Parameters for synthesis of the time-controlled *f*-Cu samples, with measured current density and corresponding efficiencies.

Deposition Time (s)	Total Deposition Charge (C)	J at 1 V vs. RHE (mA cm^{-2})	Current Efficiency (%)
0	0	0.434	78.88
10	10	1.309	78.99
30	30	1.651	55.31
50	50	2.340	79.81
80	80	2.823	88.17
100	100	2.686	83.61

Table S2 Parameters for synthesis of the current-controlled *f*-Cu samples, with measured current density and corresponding efficiencies.

Deposition Time (s)	Deposition Current (mA)	J at 1 V vs. RHE (mA cm^{-2})	Current Efficiency (%)
400	200	2.261	77.55
200	400	2.724	78.99
134	600	2.525	72.33
100	800	1.961	86.81
80	1000	2.704	88.17

Table S3 Summary of the double-layer capacitance values measured through the cyclic voltammograms. Roughness factors are normalized against mirror-finished smooth Cu plate of 1 x 1 cm^2 geometric surface area.

Electrode	VSI R_q (μm)	Pore Depth (μm)	Capacitance (μF cm^{-2})	Roughness Factor (RF)
**Smooth**	0	0	6.28	1
**10 s**	0.69	n/a	30.64	4.87
**30 s**	6.60	15.5	73.02	11.61
**50 s**	11.50	28.3	118.39	18.83
**80 s**	17.35	40.9	266.62	42.40
**100 s**	25.77	42.6	327.41	52.07
**200 mA**	30.00	60.0	510.61	81.20
**400 mA**	20.00	51.4	281.77	44.81
**600 mA**	13.97	37.5	269.05	42.79
**800 mA**	13.60	32.2	290.48	65.43
**1000 mA**	17.35	40.9	266.62	42.40

Table S4 Summary of the double-layer capacitance values measured through the cyclic voltammograms. Roughness factors are normalized against the smooth Cu plate of 1 x 1 cm^2 geometric surface area

**Sample**	**Capacitance ($\mu F\ cm^{-2}$)**	**Roughness Factor (RF)**
Smooth	6.28	1
10s	30.64	4.87
30s	73.02	11.61
50s	118.39	18.83
80s	266.62	42.40
100s	327.41	52.07
200mA	510.61	81.20
400mA	281.77	44.81
600mA	269.05	42.79
800mA	411.47	65.43
1000mA	266.62	42.40
